# Retraction: Flash Therapy for Cancer: A Potentially New Radiotherapy Methodology

**DOI:** 10.7759/cureus.r201

**Published:** 2025-11-19

**Authors:** Georgiy Georgievich Polevoy, Devika S Kumar, Sushma Daripelli, Muthu Prasanna

**Affiliations:** 1 Department of Physical Education, Moscow Polytechnic University, Moscow, RUS; 2 Department of Research and Development, Saveetha Institute of Medical and Technical Sciences (SIMATS), Chennai, IND; 3 Department of Anatomy, Government Medical College (GMC) Jangaon, Jangaon, IND; 4 Department of Pharmaceutical Biotechnology, Surya Group of Institutions, Tamil Nadu, IND

The Editors-in-Chief have retracted this article. An investigation by the journal found evidence of authorship manipulation. The Editors-in-Chief therefore no longer have confidence in the provenance and reliability of the article contents. The authors disagree with the decision to retract.

